# Phytochemical Profile of the Ethanol Extract of *Malvaviscus arboreus* Red Flower and Investigation of the Antioxidant, Antimicrobial, and Cytotoxic Activities

**DOI:** 10.3390/antibiotics11111652

**Published:** 2022-11-18

**Authors:** Hanaa S. S. Gazwi, Nagwa A. Shoeib, Magda E. Mahmoud, Osama I. A. Soltan, Moaz M. Hamed, Amany E. Ragab

**Affiliations:** 1 Department of Agricultural Chemistry, Faculty of Agriculture, Minia University, El-Minya 61519, Egypt; 2 Department of Pharmacognosy, Faculty of Pharmacy, Tanta University, Tanta 31257, Egypt; 3 Department of Food Science, Faculty of Agriculture, Minia University, El-Minia 61519, Egypt; 4 Marine Microbiology Laboratory, National Institute of Oceanography and Fisheries, Cairo 11562, Egypt

**Keywords:** *Malvaviscus arboreus*, GC-MS, HPLC, antimicrobial, antioxidant, HepG2

## Abstract

Flowers are rich sources of bioactive antimicrobial, antioxidant, and anticancer components. This study aimed to determine the constituents of the ethanol extract of *Malvaviscus arboreus* red flower (ERF) by GC-MS analysis and HPLC identification of phenolic compounds and flavonoids, in addition to the ^1^HNMR fingerprint. The antimicrobial, antioxidant, and cytotoxic activities of the ERF were investigated. The GC-MS analysis revealed twenty-one components, while HPLC analysis revealed the presence of phenolic and flavonoid compounds. The ERF showed antifungal and antibacterial activity. The highest antibacterial activity was found against *Vibrio damsela* where a time-kill assay revealed a decline in the amount of viable *V. damsela*. For fungi, the highest activity was observed against *Aspergillus terreus*. Using the SRB test on HepG2, the anti-proliferative efficacy of the ERF was evaluated. Cell cycle analysis was utilized to determine autophagic cell death. The ERF prevented the proliferation of the HepG2 cell line with an IC_50_ of 67.182 µg/µL. The extract primarily promoted apoptosis in HepG2 cells by accumulating hypodiploid cells in the sub-G0/G1 phase, increased caspase 3/7 activity, and caused considerable autophagic cell death in apoptosis-deficient cells. Finally, the observed elevation of cancer cell death indicated that ERF had substantial anticancer potential against HepG2 cells.

## 1. Introduction

Cancer is one of the biggest causes of mortality in the world, accounting for an estimated 9.9 million lives lost in 2020 [1]. A recent study found that hepatocellular carcinoma (HCC) was the fourth greatest cause of cancer-related fatalities worldwide [2]. The prognosis for this form of cancer is dismal [3], as it is typically diagnosed late. Unlike several other cancers, these strike more frequently in developing nations. HCC usually happens alongside cirrhosis, which can be caused by the hepatitis C virus, hepatitis B virus, alcoholism, Wilson’s disease, type 2 diabetes, hemochromatosis, and hemophilia. Still, the hepatitis B virus and hepatitis C virus are the main causes of liver cancer [4]. Previous studies indicated that oxidative stress plays a role in liver cancer [5], but its mechanisms and impacts remain unclear. Reactive oxygen species (ROS) such as superoxide anion (O_2_^−^), hydrogen peroxide (H_2_O_2_), and hydroxyl radical (HO), which are mostly made by breathing, inflammation, or metabolism, can cause mutations or lesions in larger genomic sites. In addition, H_2_O_2_ is a signaling molecule that balances inflammation, separation, growth, protection, metastasis, autophagy, division, and metabolic pathways. In cancer, the activity of these pathways is a key determinant of malignancy [6]. Antioxidants and peroxidants are kept in balance in check within a healthy cell. Oncogenesis and tumor growth in HCC are triggered by an imbalance of peroxidants and antioxidants [7]. Chemotherapeutic drugs are currently limited in treating cancer due to side effects and tumor resistance. New and safe anticancer drugs can be found in natural sources [8].

Antimicrobial resistance is a worldwide problem urging the research for new pipelines of natural or synthetic sources. Therefore, it is a significant challenge to find innovative and safe therapeutic choices [9].

Bioactive compounds are abundant in plant extracts. This is due to a range of chemical ingredients, for example, alkaloids, polyphenols, and flavonoids, all of which play an essential part in the drug development process [10]. In Africa, many medicinal plants are utilized to cure different illnesses since they are a viable option, especially in developing countries. *Malvaviscus arboreus* Cav. is a tropical and subtropical perennial deciduous shrub endemic to Central and South America. This plant has multiple common names, encompassing Wax mallow, Drummond Wax Mallow, Turk’s cap, and Sleeping Hibiscus. The leaves of *M. arboreus* contain compounds such as protocatechuic acid, chlorogenic acid, gallic acid, *p*-coumaric acid, ferulic acid, and hydroxybenzoic acid [11]. *Malvaviscus arboreus* has been used in traditional medicine. The leaf decoction is used for cystitis, diarrhea, fever, and gastritis [12]. The flower decoction is used as a gargle for sore throat, nursing infants with cold, bronchitis, diarrhea, thrush, and tonsillitis.

There is no information on the chemical components and the biological effects of the ethanol extract of red flowers (ERF) of *M. arboreus* in the literature. The current study investigated chemical constituents of ERF of *M. arboreus*, and its antimicrobial and antioxidant effects, in addition to the cytotoxic action on the HepG2 cell cline.

## 2. Materials and Methods

### 2.1. Preparation of the Extract

The *Malvaviscus arboreus* red flowers were collected from the campus of Minia University in May 2021 and authenticated by Professor Raga A. Taha, Horticulture Department, Faculty of Agriculture, Minia University. The flowers were washed with distilled water and kept at room temperature to dry. The dry flowers were ground and soaked in ethanol (100 mL ethanol for a 10 g dry sample) at room temperature for 24 h, filtered through Whatman No.4 filter paper (Whatman^®^Prepleated Qualitative Filter Paper, Grade 4 V, Sigma-Aldrich Company Ltd. (St. Louis, MO, USA)), and the extract was evaporated using a rotary evaporator (Büchi Rotavapor R-114 a Waterbath. B-480, Buchi, Switzerland) at 40 °C to obtain the crude extract. Then the extract was kept at 4 °C until used in the analysis.

### 2.2. Phytochemical Examination

The presence of coumarins, saponins, tannins, flavonoids, glycosides, phenols, steroids, terpenoids, emodins, anthocyanins, and alkaloids in the ERF of *M. arboreus* was investigated using qualitative assays as previously reported [13].

### 2.3. Determination of Total Phenolic Content (TPC) and Total Flavonoid Content (TFC)

As previously reported [14], total flavonoid content (TFC) in the ERF of *M. arboreus* was examined using aluminum chloride (AlCl_3_) colorimetric test. The flavonoid concentration was calculated as mg of quercetin equivalent/g extract. The total phenolic content (TPC) in the ERF of *M. arboreus* was recorded by the Folin–Ciocalteu assay [14]. The amount of phenolics was calculated as mg of gallic acid equivalent/g extract.

### 2.4. Antioxidant Activities (ABTS+, FRAP, DPPH, Metal Chelating Property, and ORAC)

Different assays were utilized to evaluate the antioxidant potential of the ERF of *M. arboreus*. The radical scavenging activity of 2,2-azinobis 3-ethylbenzothiazoline-6-sulfonic acid (ABTS+) and 1,1-diphenyl-2-picrylhydrazyl (DPPH), in addition to the ferric reducing antioxidant power (FRAP) were measured following the procedures published by Adedapo et al. [15]. The capacity of the extract to chelate iron (II) was evaluated using the procedure described by Gülc et al. [16]. For the metal chelating activity test, the ORAC assay was performed as published by Ou et al. [17].

### 2.5. GC-MS Analysis

The GC-MS analysis of ERF of *M. arboreus* was performed following a published procedure [18] using a Trace GC1310-ISQ mass spectrometer (Thermo Scientific, Austin, TX, USA) with a direct capillary column TG–5MS (30 mm × 0.25 mm × 0.25 μm film thickness, Thermo Scientific, Austin, TX, USA). The column’s oven temperature was kept at 50 °C; following that, it was set to reach 200 °C at 7 °C/min, held for 2 min, and then set to reach 290 °C, increased at 15 °C /min and maintained for 2 min. The temperature in the injector was kept at 260 °C. At a steady flow rate of 1 mL/min, helium was utilized as the carrier gas. After a 4-min solvent delay, an AS3000 autosampler and GC in the split mode were employed to automatically inject 1 μL of the diluted sample. At 70 eV ionization voltages spanning the m/z 50–650 range, EI mass spectra were acquired in full scan mode. The ion source and transfer line were adjusted to 270 and 250 degrees, respectively. By contrasting the components’ mass spectra retention times to the NIST 11 and WILEY 09 mass spectral databases, the components were identified.

### 2.6. HPLC Determination of Phenolics and Flavonoids

The phenolic and flavonoid components of the ERF were determined using an injection volume of 25 μL of the extract as previously reported [19] in an HPLC system (Agilent 1100; Santa Clara, CA, USA).

The extract phenolic components were identified using an HPLC system (Agilent 1100; Santa Clara, CA, USA) with a UV/Vis detector at a wavelength of 250 nm using a C18 column (125 × 4.60 mm, particle size 5 µm). The Agilent Chem Station was used to acquire and analyze chromatograms. To completely separate the components of phenolic acids, a mobile gradient phase of two solvents methanol [A] and acetic acid in water (1:25) [B] was used. The gradient program started at 100% B and stayed for the first three min. This was followed by 5 min of 50% eluent A, 2 min of 80% A, 5 min of 50% A, and the detection wavelength was at 250 nm.

The same HPLC system was used to identify the flavonoid components in the extract using a C18 column (Agilent; Santa Clara, CA, USA) (250 × 4.6 mm, 5 µm) and a UV/Vis detector at a wavelength of 360 nm. Acetonitrile (A) and 0.2% (*v*/*v*) aqueous formic acid (B) were used as the mobile phase with an isocratic elution (70:30) procedure.

### 2.7. ^1^HNMR Fingerprint Analysis

The ^1^HNMR fingerprint was analyzed at 400 MHz using a Bruker Avance 400 spectrophotometer (Karlsruh, Germany), using DMSO as a solvent and tetramethylsilane (TMS) as an internal standard.

### 2.8. Antibacterial Activity

#### 2.8.1. Test Microorganism

The bacterial strains *Enterococcus faecalis*, *Bacillus subtillus*, *Bacillus cereus*, *Staphylococcus aureus*, *Vibrio fluvialis*, *Vibrio damsela*, *Pseudomonas aeruginosa*, and *Salmonella typhimurium*; and the *fungal strains Aspergillus fumigatus*, *Aspergillus terreus*, *Aspergillus niger*, *Aspergillus flavus*, *Aspergillus parasiticus*, and *Penicillium oxalicum* used in this work were provided by the Department of Microbiology, National Institute of Oceanography and Fishers, Red Sea branch, Egypt. These strains were isolated from marine sources and identified by Dr. Moaz M. Hamed. The strains were kept at 2 °C on nutrient agar slants for bacteria and Potato Dextrose Agar (PDA) (Neogen Corporation, Lansing, MI, USA) for fungi slants. The slants were folded with 25% glycerol to ensure long-term preservation.

#### 2.8.2. Bacterial Inactivation by ERF

The agar well diffusion assay technique was used to measure antibacterial activity. Antibacterial susceptibility assay of ERF was performed against the selected pathogens. In a petri-dish containing 20 mL of Muller Hinton agar media (composed of g/L is: beef ex-tract 2.0; acid hydrolysate of casein 17.5; Starch 1.5 and agar 17.5), the agar plate surface was inoculated by spreading a volume of the microbial inoculum (0.1 mL of bacterial suspension containing 10^6^ CFU/mL) over the entire agar surface. Then, a hole with a diameter of 8 mm was punched aseptically with a sterile cork borer, and a volume of ERF (100 µL) was introduced into the well. In agar wells of control plates, we applied DMSO (0.5%) (Was purchased from R&M Marketing, Essex, UK) as a negative control and amoxicil-lin/clavulanic acid (20/10 mcg) as a positive control, and then incubated the plates at 37 °C for 24 h [20].

#### 2.8.3. Minimum Inhibitory Concentration (MIC)

A tetrazolium microplate assay was used to determine the minimum inhibitory concentrations (MICs) of the test organisms [21]. A 96-well clear microtiter plate was used for the experiment. Each well of the 96-well plate was inoculated with a suspension of freshly isolated bacteria (0.1 mL) at a concentration of 5 × 10^5^ CFU/mL. Different concentrations, 15 to 0.25 mg/mL, of the test extract were diluted in series with Muller–Hinton broth (Becton Dickinson, Sparks, MD, USA). A volume of 200 µL of each concentration was added in triplicate to the wells and the plates were then incubated for 18–24 h at 37 °C ± 0.5. After incubation, in each well, 50 µL of 3-(4, 5-dimethylthiazol-2-yl)-2, 5-diphenyltetrazolium bromide (MTT), with a concentration of 0.2 mg/mL, was added and the plate was incubated at 37 °C for 30 min. The bacterial suspension without extract served as the positive control, while the corresponding solvent blank (DMSO) served as the negative control. The percentage reduction of the dye (representing the inhibition of bacterial growth) was determined by measuring the absorbance at 570 nm relative to a reference wavelength of 650 nm, which was accomplished by introducing DMSO to the spectrophotometer [22].

#### 2.8.4. Fungal Inactivation by ERF

The minimum inhibitory concentration (MIC) technique employing diffusion discs was used to evaluate antifungal activity. ERF was diluted to 25% in DMSO, followed by different concentrations of ERF (0.5–2.0 mg/mL). The strains (0.2 mL spore suspension (10^6^ spores per mL) of the tested fungal isolate) of *A. fumigatus*, *A. terreus*, *A. niger*, *A. flavus*, *A. parasiticus*, and *P. oxalicum* were activated for 24 h in a liquid culture medium, Czapek Dox broth (composition (g/L): Sucrose: 30; NaNO_3_: 3; KH_2_PO_4_: 1; MgSO_4_∙7H_2_O: 0.5; KCl: 0.5 and FeSO_4_∙7H_2_O: 0.01), at a temperature of 25 °C, and then brought to a concentration of 0.5 McFarland by spectrophotometric reading. Czapek Dox agar was used to inoculate petri dishes with the fungal strains that had already been produced. Six-millimeter sterile discs were set atop the culture medium, and 10 μL of the diluted extract was pipetted onto each one. The cultures were incubated at 25 °C for 72 h. The MIC of ERF was defined as the lowest concentration that effectively suppressed fungal growth. DMSO was used as a negative control [23].

#### 2.8.5. Time-Kill Assay

According to the preliminary findings, the ERF of *M. arboreus* had the highest level of antimicrobial activity on marine *V. damsela*. An investigation on the bactericidal effects of the ERF on *V. damsela* was carried out utilizing a time-kill test. A bacterial culture (5 × 10^6^ CFU/mL) was added to Mueller Hinton broth (MHB) containing the extract at 4 × MIC, 2 × MIC, MIC ½ × MIC, and ¼ × MIC, and untreated cultures were incubated at 37 °C. Tryptic soy agar (TSA) plates were used to culture samples for 0, 2, 4, 6, 8, 10, 12, and 24 h. A control incubation was performed with 1% DMSO. Surviving colony bacteria were counted, and log_10_ CFU/mL was calculated. A time-kill curve was analyzed by plotting log CFU/mL against time (min) [24].

#### 2.8.6. Synergistic Activity

The ERF was tested in conjunction with amoxicillin/clavulanic acid using the standard disc diffusion method against selected marine *V. damsela*. The antibacterial activity was evaluated on an agar plate using discs made by combining amoxicillin/clavulanic acid (20/10 mcg) with different doses of ERF (250, 500, 750, and 1000 µg/mL). The antibacterial effectiveness of the ERF and amoxicillin/clavulanic combination was evaluated by measuring the size of the zone of inhibition after 24 h of incubation at 37 °C [25].

### 2.9. Cytotoxic Study

#### 2.9.1. Cell Lines

Nawah Scientific Inc. provided HepG2: Hepatocellular carcinoma (Mokatam, Cairo, Egypt). In a humidified 5% (*v*/*v*) CO_2_ atmosphere, cells were kept at 37 °C in Dulbecco’s Minimum Essential Medium (DMEM, Lonza, Basel, Switzerland) media enriched with 100 units/mL penicillin, 100 mg/mL streptomycin, and 10% heat-inactivated fetal bovine serum (FBS).

#### 2.9.2. Cytotoxicity Assay

The cell viability was measured utilizing the Sulforhodamine B (SRB) test. One hundred microliter cell suspension (5 × 10^3^ cells) aliquots were incubated in complete media for 24 h in 96-well plates. A further aliquot of 100 μL media comprising ERF in varying amounts was administered to the cells. The cells were fixed after 72 h of ERF treatment by changing the medium with 150 μL of 10% Trichloroacetic acid (TCA) and incubated for 1 h at 4 °C. The cells were rinsed five times with distilled water after the TCA solution was removed. Seventy microliters of SRB solution (0.4% *w*/*v*) was administrated in aliquots and incubated for 10 min in the dark at room temperature. Before being air-dried overnight, plates were washed thrice in 1% acetic acid, then 150 μL of TRIS (10 mM) was administrated to disperse the protein-bound SRB dye, the absorbance was observed at 540 nm utilizing the FlUOstar Optima Microplate Reader (BMG LABTECH, Ortenberg, Germany).

#### 2.9.3. Analysis of Cell Cycle Distribution

A previously published procedure was followed for the analysis of the cell cycle distribution [26]. One hundred and five cells were trypsinized and rinsed twice with ice-cold phosphate buffered saline (PBS) after being treated with test drugs for 24 or 48 h and paclitaxel (1 µM) for 24 h as a positive control (pH 7.4). The cells were fixed by resuspending them in 2 mL of 60% ice-cold ethanol and incubating them for one h at 4 °C. After being rinsed twice with PBS, the fixed pellet was resuspended in 1 mL of PBS (pH 7.4) with 50 µg/mL RNAase A and 10 µg/mL propidium iodide (PI). An FL2 (λex/em 535/617 nm) signal detector was used to determine the DNA content of cells after 20 min of incubation in the dark at 37 °C. (ACEA Novocyte^TM^ flow cytometer, ACEA Biosciences Inc., San Diego, CA, USA). Each specimen was made up of 12,000 events in total. The ACEA NovoExpress application was used to calculate the cell cycle dispersion (ACEA Biosciences Inc., San Diego, CA, USA).

#### 2.9.4. Apoptosis Assay

Flow cytometry with two fluorescent channels and an annexin V-FITC apoptosis detection kit were employed to identify apoptosis and necrosis in cell populations (Abcam Inc., Cambridge Science Park, Cambridge, UK) using a published procedure [26]. After 24/48 or 72 h of treatment with ERF and doxorubicin (10 µM) as a positive control, cells (10^5^) were trypsinized and rinsed twice with ice-cold PBS (pH 7.4). The cells were then maintained at room temperature in the dark for 30 min with Annexin V-FITC/PI solution 0.5 mL, as directed by the manufacturer. After labeling, cells were added to an ACEA Novocyte^TM^ flow cytometer (ACEA Biosciences Inc., San Diego, CA, USA) and measured for PI and FTIC fluorescent signals with FL1 and FL2 signal detectors (λ_ex_/_em_ 488/530 nm for FITC and λ_ex_/_em_ 535/617 nm for PI, respectively). ACEA NovoExpress^TM^ software was used to assess the positive FITC or PI cells for each sample, utilizing quadrant analysis (ACEA Biosciences Inc., San Diego, CA, USA).

#### 2.9.5. Autophagy Assay

Autophagic cell death was measured by flow cytometry and acridine orange lysosomal staining. A total of 10^5^ cells were trypsinized and rinsed twice with ice-cold PBS after treatment with ERF for 24/48 or 72 h and chloroquine (10 µM) as a positive control for 24/48 or 72 h (pH 7.4). The cells were stained with acridine orange (10 µM) and incubated for 30 min at 37 °C in the dark. The acridine orange fluorescence signals using an FL1 signal detector (λ_ex_/_em_ 488/530 nm) in an ACEA Novocyte^TM^ flow cytometer (ACEA Biosciences Inc., San Diego, CA, USA). The ACEA NovoExpress^TM^ software was used to calculate net fluorescence intensity (NFI) from 12,000 incidences per specimen (ACEA Biosciences Inc., San Diego, CA, USA).

#### 2.9.6. Caspase-Glo 3/7 Activity

The impact of the IC_50_ of ERF on caspase 3/7 activity in HepG2 cells was evaluated using the Caspase-Glo 3/7 Assay kit (Promega, Walldorf, Germany), according to the manufacturer’s instructions. Caspase activity was expressed as a proportion of the untreated control [27].

### 2.10. Statistical Analysis

The Graphpad Prism 6 software was used to conduct all statistical analyses. A one-way analysis of variance was utilized to compare the results (ANOVA). The statistical significance was determined as a *p*-value < 0.05.

## 3. Results and Discussion

Plant extracts have substantial therapeutic potential with few negative adverse effects for treating infectious diseases, making medicinal herbs an appealing source of new medicinal components. The therapeutic potentials are related to the phytochemical components. The phytochemical profile ERF of *M. arboreus* was investigated. 

### 3.1. Phytochemical Evaluation of ERF of M. arboreus

#### 3.1.1. Phytochemical Screening

The preliminary screening of ERF of *M. arboreus* showed the presence of many phytoconstituents, for example flavonoids, tannins, coumarins, saponins, glycosides, phenols, terpenoids, steroids, emodins, alkaloids, and anthocyanins, which might account for their medicinal effects (Table 1).

#### 3.1.2. Total Flavonoid and Phenolic Contents

The ERF of *M. arboreus* showed TFC and TPC of 23.83 ± 2.9 mg quercetin equivalent/g extract and 46.25 ± 2.1 mg gallic acid equivalent/g extract, respectively, as well as high antioxidant activity (Table 2). These phytoconstituents were shown to have a variety of therapeutic activities and were known to be biologically active compounds [28].

#### 3.1.3. GC/MS Analysis

**Figure 1:**
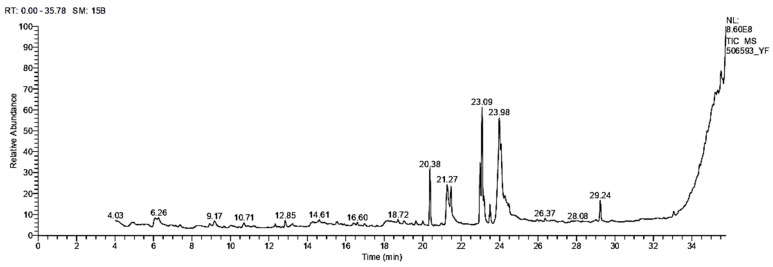
GC-MS chromatogram of the ERF of *M. arboreus*.

Figure 1 shows a total scan gas chromatogram of the ERF of *M. arboreus*. It demonstrated the presence of several bioactive chemicals with varying retention times (RT). Table 3 shows the molecular weight, RT, and percent peak area, as well as chemical formulae of the identified compounds. Additionally, the biological functions of the identified compounds, as anticipated by Dr. Duke’s phytochemical and ethnobotanical databases (USDA, Agricultural Research Service, 1992–2016), are also summarized in Table 3.

In the ERF of *M. arboreus*, 21 components were found. The predominant compounds were 11-octadecenoic acid methyl ester (19.49%), 9,12-octadecadienoic acid (Z, Z)-2-hydroxy-1-(hydroxymethyl)ethyl ester (18.41%), 9,12-Octadecadienoic acid (Z, Z)-methyl ester (9.46%), hexadecanoic acid methyl ester (8.88%), hexadecanoic acid (8.52%), and oleic acid (7.73%).

#### 3.1.4. Identification and Quantification of Phenolics and Flavonoids

**Figure 2:**
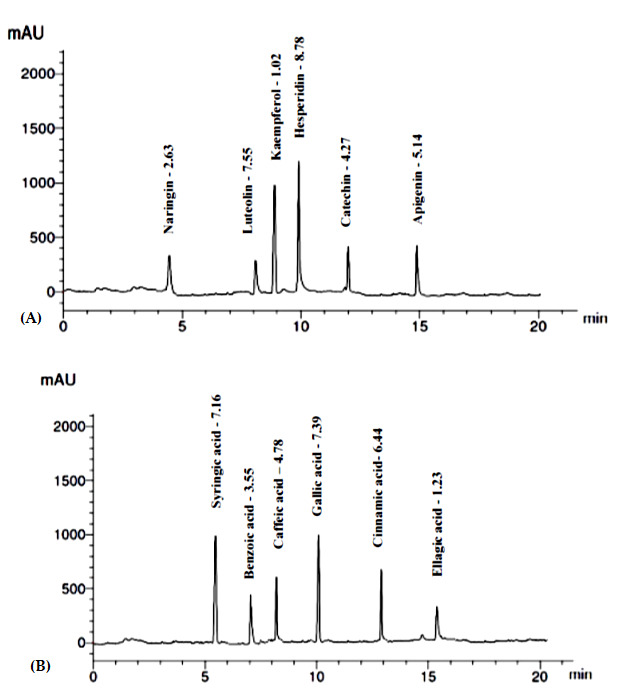
HPLC chromatogram showing identified flavonoids (**A**) and phenolic acids (**B**) in the ERF.

HPLC examination of the ERF of *M. arboreus* revealed the identification and quantification of 13 polyphenolic compounds (6 flavonoids and 7 phenolic acids), as shown in Table 4. The compounds were identified by comparison to authentic samples analyzed using the same procedures. Hesperidin and luteolin were the major flavonoids identified at concentrations of 8.78 and 7.55 µg/mg of the ERF. Gallic acid was the predominant phenolic component identified in the extract (7.39 µg/mg), followed by syringic acid (7.16 µg/mg) and cinnamic acid (6.44 µg/mg) (Figure 2, Table 4).

#### 3.1.5. ^1^HNMR Fingerprint of the ERF

**Figure 3:**
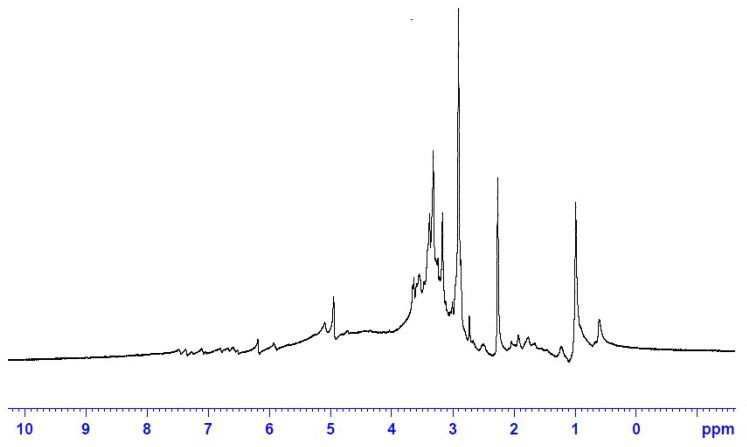
^1^HNMR spectrum of the ERF of *M. arboreus* in DMSO (400 MHz).

The ^1^HNMR spectrum of the ERF at 400 MHz (Figure 3) revealed that the extract is rich in oxygenated saturated and unsaturated hydrocarbon compounds. Signals in the range 0.0–4.0 ppm are predominant in the spectrum while signals in the aromatic range 6.0–8.0 ppm are weak. In correlation to the GC and HPLC analysis, the extract is rich in fatty acids (saturated and unsaturated), which explains why the fingerprint pattern as the characteristic signals for fatty acids are 2.0–2.5 ppm for (CH_2_), 3.0–4.0 ppm for (-CHOH-, CH_3_-CO-, -CH_2_-CO-), 5.0–6.0 ppm (-CH=CH-) [29].

### 3.2. Antioxidant Capacities of ERF of M. arboreus

The equivalent antioxidant capacities of trolox (TE) as compared to the ERF were 716.45 ± 16.12, 99.15 ± 4.96, 1138.11 ± 79.65 µM TE/mg extract in ABTS, FRAP, and ORAC assays, respectively. ERF exerted high free radical scavenging activity against DPPH radical (IC_50_ = 115.6 ± 16.9 μg /mL) and high ability of its metal chelating property (57.58 ± 3.5 µM EDTA eq/mg extract) (Table 5).

Antioxidant properties are well known in phenolic compounds by acting as reducing agents, free radical scavengers, or metal chelators [30]. The most abundant plant phenolics are flavonoids and phenolic acids, which have a substantial antioxidant activity both in vitro as well as in vivo [31].

### 3.3. Antibacterial and Antifungal Activities of Extract 

In many regions of the world, there is a great deal of interest in medicinal plants as therapeutic medications because of the rise in drug-resistant bacteria and the emergence of more pathogenic bacterial species. Many medicinal plants have been studied in vitro against bacterial strains, with extracts and pure components of several medicinal plants being particularly beneficial [32].

Eight different strains of marine pathogenic bacteria were selected in this study, including *B. subtillus*, *E. faecalis*, *B. cereus*, *S. typhimurium*, *P. aeruginosa*, *V. fluvialis*, *S. aureus*, and *V. damsela*. The ERF of *M. arboreus* showed an antibacterial effect against most of the tested strains with average inhibition zones ranging between 10 and 20 mm compared to the positive control amoxicillin/clavulanic acid (Table 6). The ERF of *M. arboreus* exhibited a strong antibacterial activity against *V. damsela* with an inhibition zone of 20 ± 0.2 mm, moderate antibacterial activity against *V. fluvialis* and *S. typhimurium* with the inhibition zones being 16 mm, and showed lower effects against *E. faecalis*, *S. aureus*, and *P. aeruginosa* with inhibition zones of 10, 12, and 14 mm, respectively. On the other hand, the ERF of *M. arboreus* was ineffective against *B. subtilus* and *B. cereus*. The negative control (DMSO) showed no zone of inhibition.

In our study, the ERF presented activity against *A. terreus*, *A. fumigatus*, and *A. flavus* respectively, with no effect on the other strains. The ERF of *M. arboreus* did not show any effect on either *A. parasiticus* and *P. oxalicum*. To establish the susceptibilities of ERF against the tested strains, the minimum inhibitory concentration (MIC) values were determined (Table 7). The ERF of *M. arboreus* exhibited the lowest MIC for *V. damsela* (1.5 ± 0.02 mg/mL). The MIC values for *E. faecalis*, *S. aureus*, *P. aeruginosa*, *V. fluvialis,* and *S. typhimurium* were 12.5 ± 0.02, 10.0 ± 0.06, 10.0 ± 0.01, 2.5 ± 0.05, and 5.0 ± 0.01 mg/mL, respectively. On the other hand, the MIC values against *A. fumigatus*, *A. flavus*, *A. niger*, and *A. terreus* were 1.0 ± 0.02, 1.25 ± 0.01, 1.75 ± 0.06, and 0.75 ± 0.01 mg/mL, respectively.

#### 3.3.1. Bacterial Killing Kinetics Assay of ERF against Marine *V. damsela*

**Figure 4:**
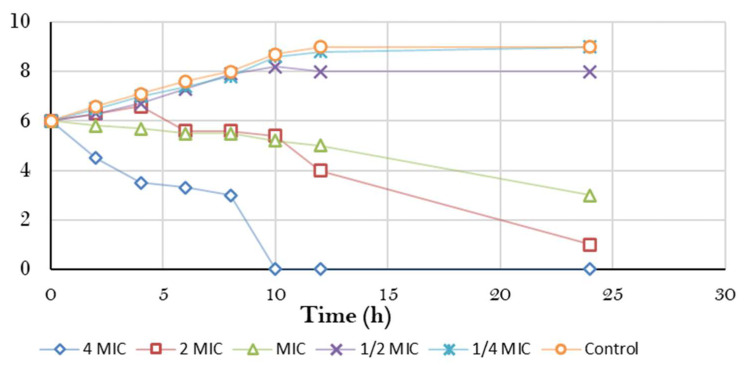
Time-kill curve of *V. damsela* by *M. arboreus* extract.

A time-kill kinetic assay of the ERF against marine *V. damsela* was investigated, with the results demonstrated in Figure 4. As a result, time-kill curve was plotted between the logarithmic number of CFU/mL and incubation time. At 4 × MIC concentration, the ERF showed a decrease in the amount of viable *V. damsela* at 8–24 h. The extent by which bacteria was inhibited by the plant extract by time varied greatly, as shown by killing analyses [33]. Therefore, the capacity of plant secondary metabolites to possess antibacterial characteristics may be taken into consideration, as well as their response to microbial infection [34].

#### 3.3.2. Analysis of the Synergistic Impact of ERF

**Figure 5:**
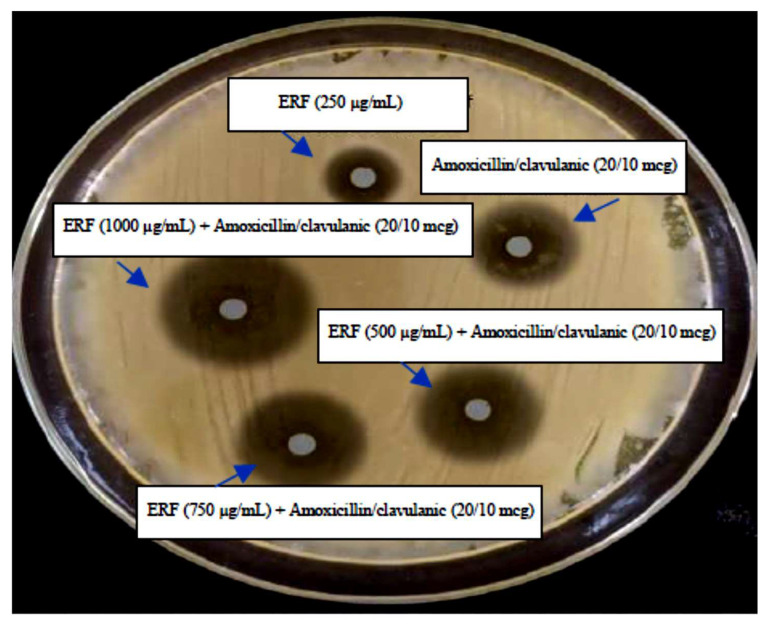
Synergistic effect of deferent concentrations of ERF and amoxicillin/clavulanic (20/10 mcg) acid against selected pathogen marine *V. damsela*.

Figure 5 and Table 8 display the findings of an evaluation of the synergistic effect of ERF and amoxicillin/clavulanic acid against the selected pathogen marine *V. damsela*. Amoxicillin/clavulanic acid at a concentration of (20/10 mcg) demonstrated moderate effective action against the *V. damsela* that were examined. When compared to 250 and 750 µg/mL, the antibacterial activity displayed by the combined effect of antibiotics and ERF was significantly stronger against the selected pathogen at a concentration of 1000 µg/mL, with a zone of inhibition ranging in diameter from 26 ± 0.2 to 28 ± 0.1 mm (Table 6). Due to the synergistic action of the ERF and amoxicillin/clavulanic, it was hypothesized that this combination therapy would be successful against the *V. damsela* that were tested.

The chemical composition of the ERF of M. arboreus revealed the existence of noteworthy chemicals such as octadecenoic acid methyl ester, hexadecanoic acid, oleic acid, 11-octadecenoic acid, and octadecanoic acid (Table 3). These compounds have proven antimicrobial activity. Cinnamic acid and its hydroxylated derivatives demonstrated antifungal properties, reducing antityrosinase enzyme activity and fungal spore germination [35]. Cinnamic acids suppressed fungal expansion via interacting with the enzyme benzoate 4-hydroxylase, which is involved in the detoxification of aromatic compounds [36]. Hexadecanoic acid reacted with the lipopolysaccharides’ hydroxyl group, an element of the bacterial cell wall, causing the lipopolysaccharide membrane structure’s asymmetric conversion, as per Johannes et al. [37]. Therefore, the lipid structure of the membrane was disrupted. The cell swelled, the cytoplasm membrane was damaged, and the cell was distended and lysed due to the alteration in the cell membrane. The hydroxyl group of hexadecanoic acid has been noticed to be toxic to the cell protoplasm, as the compound permeates the cell wall and causes damage [38].

*V. damsela* is one of the pathogens associated with infections caused by seafood; thus, the ERF may be an option for treating this infection.

### 3.4. Cytotoxic Activity

This study aims to examine the impacts of the ERF on liver cancer in vitro, utilizing the most common cell line for hepatotoxicity and drug metabolism studies, hepatocellular carcinoma HepG2. HepG2 cells are nontumorigenic, increase rapidly, have an epithelial-like shape, and are capable of performing a wide variety of differentiated liver activities [39].

**Figure 6:**
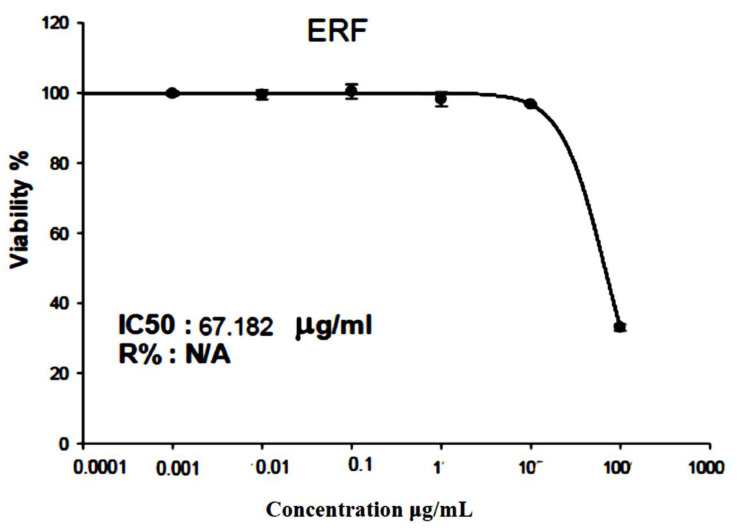
SRB assay on HepG2 cells to validate the ERF impact on the cell viability after 72 h.

**Figure 7:**
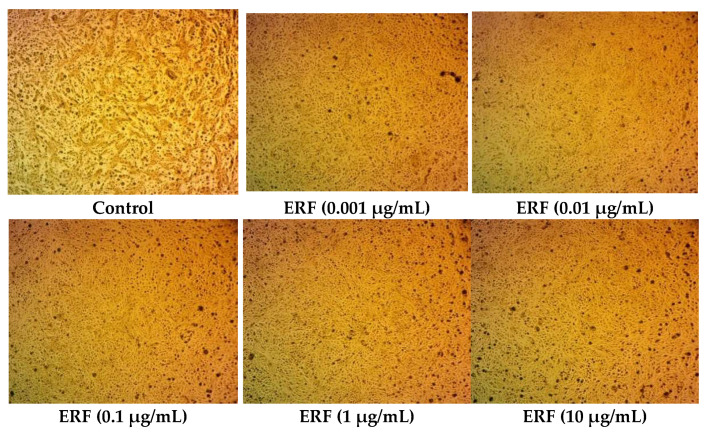

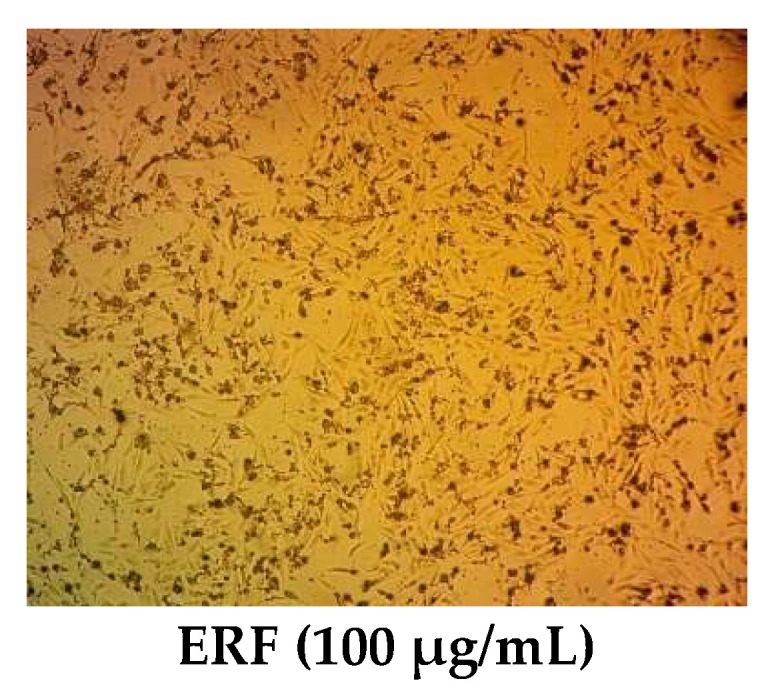
Photomicrographs of HepG2 cells treated with ERF (100× magnification).

After 72 h of incubation, the SRB test was utilized to analyze the cytotoxicity of the ERF on the hepatocellular carcinoma (HepG2) cell line. The results showed that ERF significantly reduced HepG2 cell proliferation in a dose-dependent manner, with an IC_50_ value of 67.182 µg/µL (Figure 6 and Figure 7).

Phenolics, which come in various types, are known for causing apoptosis and cytotoxicity in cancer cell lines. The capability of phenolic compounds to scavenge radicals and their antioxidant capabilities are primarily responsible for their anticancer effects. Hesperidin, a primary flavonoid in the extract under investigation, protected the rat liver against CCl_4_-induced oxidative stress and dysfunction linked to its antioxidant properties [40]. Hesperidin’s impact on the MCF-7 human breast cancer cells and prostate cancer cell proliferation was studied [41]. Abd El-Azim et al. [42] found that 4-hydroxybenzoic acid, a phenolic acid in excessive levels in the extract, had substantial cytotoxic action on both colon (HCT116) and liver (HepG2) cancer cell lines. Polyphenolic substances reduce mutagenesis and carcinogenesis in humans when consumed in up to 1.0 g per day from a diet rich in fruits, vegetables, and other plants [43].

#### 3.4.1. Cell Cycle Analysis

**Figure 8:**
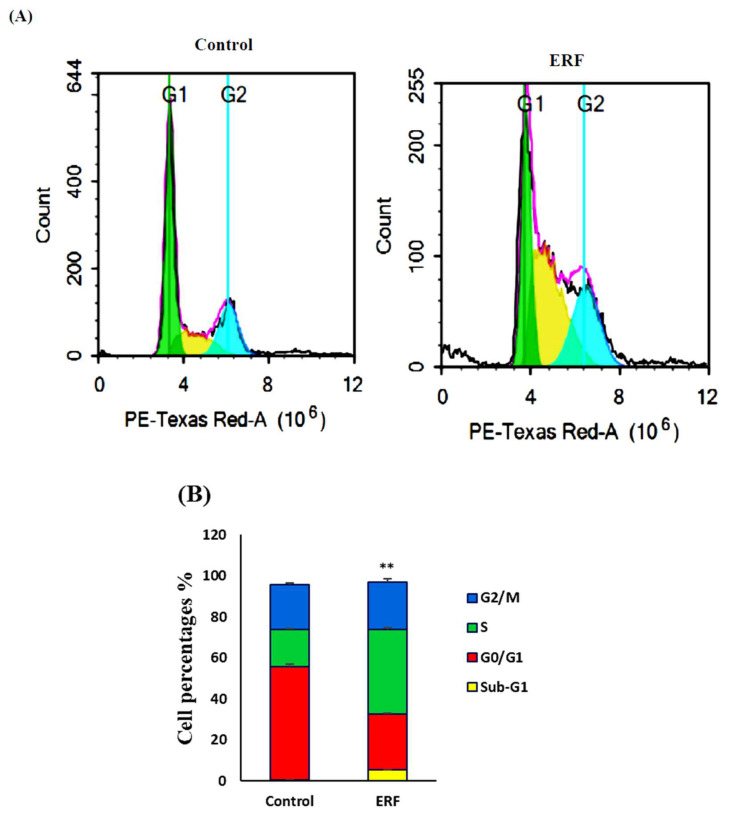
Representative flow cytometry analysis of HepG2 cells treated for 48 h with the IC_50_ of the ERF. DNA cytometry analysis was performed to examine cell cycle distribution, and diverse cell phases (**A**) were plotted as percentages of total occurrences (**B**). ** Significantly different from the control group; results are provided as mean ± SD; *n* = 3.

To explore the impact of the ERF on cell cycle distribution, HepG2 cells were treated for 48 h with the pre-determined IC_50_ of the ERF, and DNA content was measured utilizing flow cytometry. The results in Figure 8 revealed an apparent change in the distribution of different phases. In G0/G1-phase cells, ERF did not further increase antiproliferative effects (38.1 ± 1.19) compared to untreated cells (41.39 ± 0.46%).

Compared to the untreated cells (50.4 ± 3.2%), the ERF caused S-phase arrest, and thus increased the cell population (38.0 ± 1.9%). Compared to the untreated cells (0.59 ± 0.03%), ERF dramatically accelerated cell mortality as observed by an elevation in the sub-G1 phase cell population (5.31 ± 0.34%). The findings implied HepG2 cell death by exposure to the study extract.

The ERF significantly induced more cell death manifested by an increased pre-G phase cell population (5.31 ± 0.34%) compared to untreated cells (0.59 ± 0.03%). The results suggest that HepG2 cells underwent apoptosis upon treatment with the study extract.

A range of processes, including apoptosis and cell cycle arrest, were involved in the cytotoxic effects of the ERF extract. The ability of anticancer drugs to induce cell cycle arrest in cancer cells was measured [44]. A significant hypodiploid sub-G0/G1 peak was visible in the production of apoptotic cells, which was easily observed with substantial damage to cellular DNA and might be distinguished by flow cytometry [45].

According to these data, the ERF extract could produce substantial DNA loss to cause apoptosis in the present investigation, as the concentration of hypodiploid cells in the sub-G0/G1 phase was a sign of apoptotic cell death. Apoptosis would be confirmed by several intracellular pathways, such as caspase activation and MMP disruption.

#### 3.4.2. Assessing Cell Apoptosis with Annexin V-FITC

**Figure 9:**
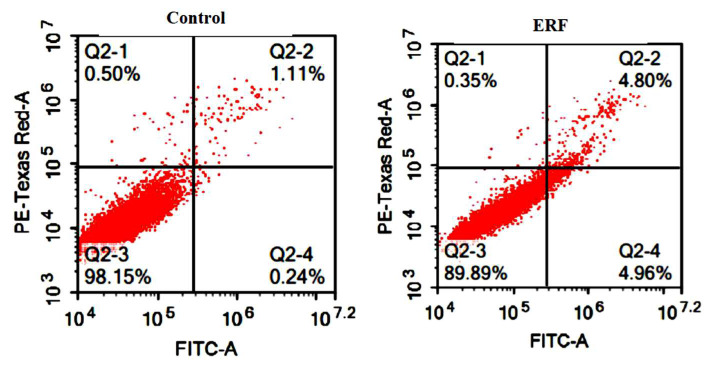

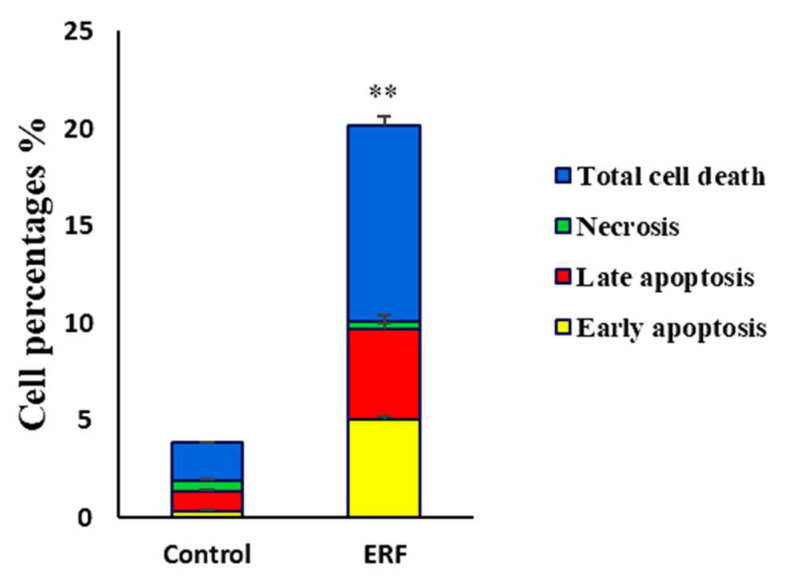
In contrast to untreated cells, apoptosis and necrosis were detected utilizing Annexin V-FITC and PI dual staining after 24 h of treating HepG2 cells with IC_50_ of the study crude extract RF. ** Significantly different from the control group; data are presented as mean ± SD; *n* = 3.

The ERF impact on the growth suppression of HepG2 cells was associated with apoptosis, as determined by apoptotic and necrotic cells’ Annexin V analysis. The cells were double-labeled with PI, which produced red fluorescence in necrotic cells, and Annexin V-FITC, which caused cytoplasmic green labeling in apoptotic cells after 24 h of treatment with the ERF extract’s IC_50_. In fluorescence microscopy images, viable cells were negative for Annexin V and PI (Figure 9). A considerable amount of green and red labeling was observed in ERF, indicating apoptotic and necrotic cells. When cells were treated with ERF, many apoptotic cells were found, indicating that this extract was primarily responsible for apoptosis.

Figure 9 demonstrates the HepG2 cells’ distribution in four quadrants (Q1 = necrosis phase, Q2 = late apoptosis, Q3 = normal intact cells, Q4 = early apoptosis phase) and represents one of three independent tests carried out. Cells that experienced apoptosis would shift from the viable quadrant (Q3) to the early apoptosis quadrant (Q4) and finally end up in the late apoptosis quadrant (Q2). Necrosis, in contrast, caused cells to move from the viable quadrant (Q3) to the late necrosis quadrant (Q2). Untreated cells had a proportion of viable cells of 98.07 ± 0.08%, dead cells of 1.93 ± 0.08%, late apoptosis of 0.37 ± 0.07%, and early apoptosis of 0.32 ± 0.09%. ERF increased the late apoptotic population to 4.67 ± 0.31%. A noticeable decrease was indicated in necrotic cells, with proportions of 0.37 ± 0.07% upon treatment, compared to untreated cells at 0.52 ± 0.12. Lastly, early apoptotic cells, expressed by Q4, demonstrated only a slight elevation in cell distribution due to treatment with the ERF to 5.05 ± 0.17%.

Phosphatidyl-serine (PS) on the outer layer of the plasma membrane served as a recognition site for phagocytes during the early stages of apoptosis [46]. Annexin V, a calcium-dependent protein, could bind to the exposed phosphatidyl-serine on the membrane’s exterior layer (PS) [47]. The percentage of cells going through late apoptosis rose exponentially in this study, indicating that apoptosis was one of the primary mechanisms in which the plant extract induced cell death in the four studies.

#### 3.4.3. Assessment of Autophagy

**Figure 10:**
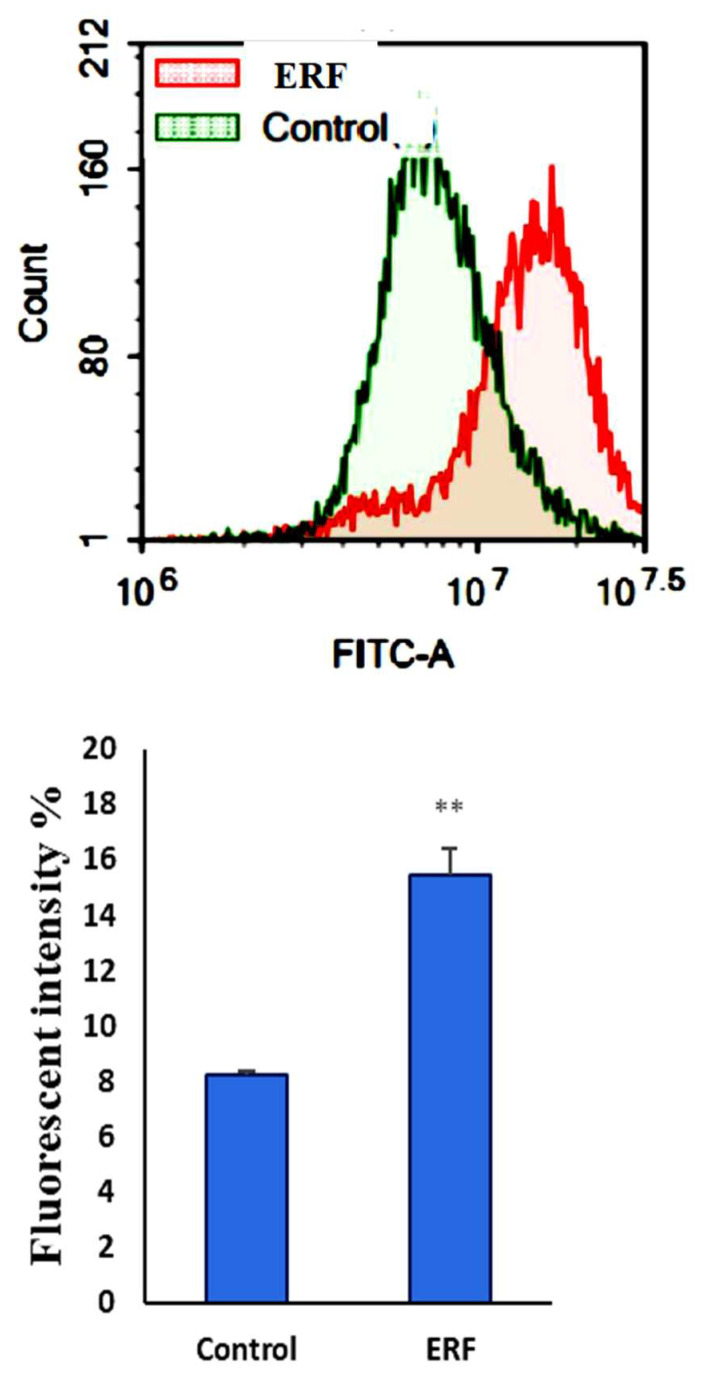
After being exposed to ERF, autophagic cell death was assessed in HepG2 cells. Cells were stained with a Cyto-ID autophagosome tracker after exposure to ERF for 24 h. ** Significantly different from the control group; data are presented as mean SD; *n* = 3.

Autophagy-mediated programmed cell death, such as apoptosis, is a significant issue in science. Using the Cyto-ID autophagy detection dye and flow cytometry, we studied the influence of ERF on the autophagy process in HepG2 cells. In comparison to the untreated cells, ERF treatment significantly boosted autophagic cell death (Figure 10).

Autophagy was another hypothesized cell death route, but its significance in cancer cell death was convoluted and controversial [48]. ERF caused considerable autophagic cell death in HepG2 cells, which could be a pro-death mechanism due to poor apoptosis in this cell type [49].

#### 3.4.4. Effect of ERF on the Activity of Caspase 3/7

The activation of caspases is required for the last step of apoptosis [50]. Understanding the stimulation route by cytotoxic substances could help model improved therapeutic options [51].

**Figure 11:**
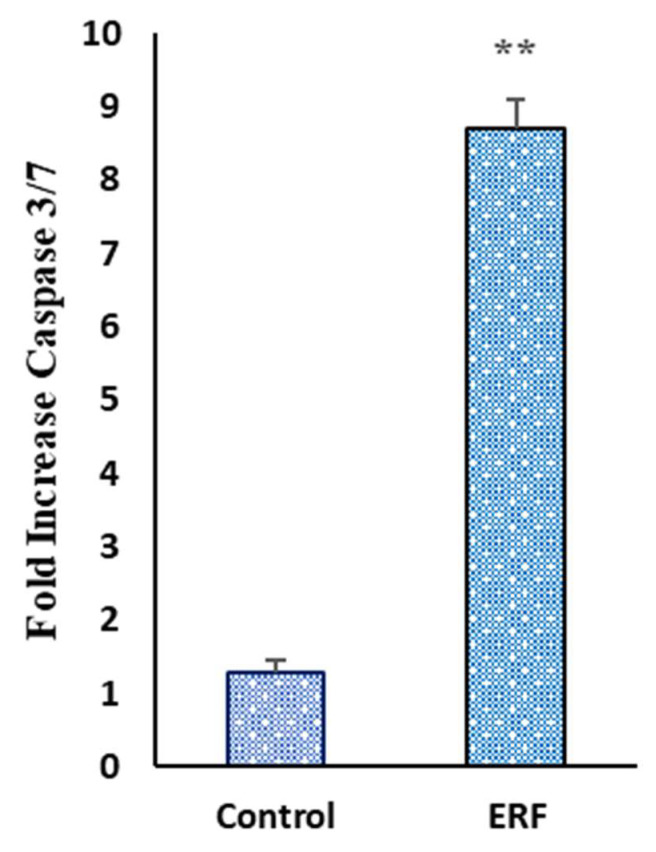
Effect of ERF on the Activity of Caspase 3/7 on HepG2 cells. All data were expressed as mean ± standard deviation (SD) at a significance level of *p* < 0.05 and indicated by **.

The caspase 3/7 activity was evaluated on HepG2 cells treated with the concentration of IC_50_ values of the extract for 24 h to determine if the apoptotic effect generated by the ERF was dependent on caspase activation (Figure 11). The activation of caspase 3/7 was increased by 8.71 ± 0.99-fold in ERF-treated cells compared to 1.28 ± 0.17-fold in the untreated cells, confirming the effect of this extract on apoptotic cell death formerly demonstrated in cell cycle studies and Annexin V. The cleavage of several caspases triggered apoptosis. Understanding the effects of caspase cleavage could help us understand cell death, as well as other biological processes [52]. The increased caspase-3 activity in the treated HepG2 suggested that extrinsic and intrinsic caspase-3 activation pathways were utilized at this dose (IC_50_). Apoptosis triggered by caspases could activate either the death receptor (extrinsic) or mitochondrial (intrinsic) pathways or both [53].

## 4. Conclusions

This is the first antimicrobial, anticancer evaluation of the ERF of *M. arboreus* against hepatocarcinoma cell line HepG2. The ERF of *M. arboreus* demonstrated anticancer effectiveness against HepG2 and an in vitro growth inhibitory impact against microbiological growth. Our GC-MS and HLPC analyses showed the existence of many phytochemical compounds that might influence the antibacterial and anticancer properties of *M. arboreus* red flower ethanolic extract. As a result, it is suggested that the antibacterial and anticancer efficacy of the GC-MS and HLPC found compounds to be evaluated to develop a novel perspective on antimicrobial and anticancer medicine and assess the mode of action used to combat anticancer recovery.

## Figures and Tables

**Table 1:** Phytochemical screening of ERF of *M. arboreus*.

Tests	Result
Flavonoids	+
Tannins	+
Coumarins	+
Saponins	±
Steroids	±
Glycosides	+
Phenols	+
Terpenoids	+
Emodins	+
Anthocyanins	+
Alkaloids	±

(+) positive test; (±) faint.

**Table 2:** Total flavonoid and phenolics contents of ERF of *M. arboreus*.

Parameters	Result
TPC (mg GAE/g extract)	46.25 ± 2.1
TFC (mg QE/g extract)	23.83 ± 2.9

Variables are shown as mean ± SD (standard deviation, *n* = 3). GAE: gallic acid equivalent; QE: quercetin equivalent.

**Table 3:** The identified compounds in the ERF of *M. arboreus* RF by GC/MS analysis.

	RT	Name of the Compound	MF	MW	Peak Area (%)	Biological Activity **
1	6.07	4H-Pyran-4-one, 2,3-dihydro-3,5-dihydroxy-6-methyl	C_6_H_8_O_4_	144	2.85	Antimicrobial, anti-inflammatory
2	6.26	Octadecanoic acid, ethyl ester	C_20_H_40_O_2_	312	1.46	Anti-microbial
3	9.17	3,5-Heptadienal, 2-ethylidene-6-methyl-	C_10_H_14_O	150	1.14	Anti-inflammatory, antitumor, antiviral
4	10.71	4-(3,3-dimethyl-1-butynyl)-4-hydroxy-2,6,6-trimethyl-2-cyclohexen-1-one	C_15_H_22_O_2_	234	1.09	Antioxidant, anti-inflammatory
5	12.33	10,13-Octadecadiynoic acid, methyl ester	C_19_H_30_O_2_	290	0.60	No activity reported
6	12.84	1-(3-Methoxy-5-methylphenyl)-*N*-methylpropan-2-amine	C_12_H_19_N_O_	193	1.20	No activity reported
7	18.72	9,12,15-Octadecatrienoic acid, 2,3-bis [(trimethylsilyl)oxy] propyl ester, (z, z, z)-	C_27_H_52_O_4_Si_2_	496	0.66	Anticancer, hepatoprotective
8	19.64	Cis-13-Eicosenoic acid	C_20_H_38_O_2_	310	0.81	Anti-inflammatory activity
9	20.01	9-octadecenoic acid, (2-phenyl-1,3-dioxolan-4-yl) methyl ester, cis	C_28_H_44_O_4_	444	0.72	Antimicrobial, anti-inflammatory
10	20.38	Hexadecanoic acid, methyl ester	C_17_H34O_2_	270	8.88	Antioxidant, antimicrobial, antihypercholesterolemic property
11	21.27	Hexadecanoic acid	C_16_H_32_O_2_	256	8.52	Anti-inflammatory, antioxidant, antihypercholesterolemic
12	21.47	Hexadecanoic acid, ethyl ester	C_18_H_36_O_2_	284	3.66	Antioxidant, antihypercholesterolemic antiandrogenic
13	22.99	9,12-Octadecadienoic acid (Z, Z)-, methyl ester	C_19_H_34_O_2_	294	9.46	Hepatoprotective, antihistamine, hypocholesterolemia, anti-eczema
14	23.09	11-Octadecenoic acid, methyl ester	C_19_H_36_O_2_		19.49	Antioxidant, antimicrobial
15	23.19	16-Octadecenoic acid, methyl ester	C_19_H_36_O_2_	296	3.86	Selectively inhibit eukaryotic DNA polymerase activities in vitro
16	23.50	Octadecanoic acid, methyl ester	C_19_H_38_O_2_	298	3.03	Antimicrobial
17	23.98	9,12-Octadecadienoic acid (Z, Z)-, 2-hydroxy-1-(hydroxymethyl)ethyl ester	C_21_H_38_O_4_	354	18.41	Antiarthritic, hepatoprotective, antiandrogenic, anticoronary, antieczemic, anticancer
18	24.08	Ethyl oleate	C_18_H_34_O_2_	282	7.73	Antibacterial, anticancer
19	24.28	Oleic acid	C_20_H_38_O_2_	310	1.25	It is used as a vehicle for intramuscular drug delivery, progesterone
20	24.41	Linoleic acid ethyl ester	C_20_H_36_O_2_	308	0.71	Anti-arthritic, anti-acne, hepatoprotective, anti-histaminic, anti-coronary
21	24.49	Octadecanoic acid, 2,3-dihydroxypropyl ester	C_21_H_42_O_4_	358	1.00	Anticancer, antimicrobial

** Dr. Duke’s Phytochemical and Ethnobotanical Databases. RT: retention time; MF: molecular formula; MW: molecular weight.

**Table 4:** HPLC analysis of phenolics and flavonoids in the ERF of *M. arboreus*.

Components	RT (min)	Conc. (µg/mg)
Flavonoid compounds		
Naringin	4.6	2.63
Hesperidin	10.0	8.78
Kaempferol	8.1	1.02
Luteolin	9.0	7.55
Apigenin	15.0	5.14
Catechin	12.0	4.27
Phenolic compounds		
Caffeic acid	8.1	4.78
Cinnamic acid	13.0	6.44
Gallic acid	10.0	7.39
Syringic acid	5.2	7.16
Benzoic acid	7.0	3.55
Ellagic acid	15. 6	1.23

**Table 5:** Antioxidant capacities of the ERF of *M. arboreus*.

Parameters	Result
DPPH (IC_50_ μg/mL)	115.6 ± 16.9
ABTS (µM TE/mg extract)	716.45 ± 16.12
FRAP (μM TE/mg extract)	99.15 ± 4.96
ORAC (µM TE/mg extract)	1138.11 ± 79.65
Metal chelating property (µM EDTA eq/mg extract)	57.58 ± 3.5

Variables are shown as mean ± SD (standard deviation, *n* = 3).

**Table 6:** Antimicrobial activity of the crude extract of red flower of *M. arboreus* using well-cut diffusion method.

Pathogens	Inhibition Zone (mm)	Amoxicillin/Clavulanic (Positive Control)	DMSO (Negative Control)
*B. subtillus*	0.0	14.0 ± 0.5	0.0
*S. aureus*	12.0 ± 0.6	12.0. ± 0.2	0.0
*E. faecalis*	10.0 ± 0.1	18.0 ± 0.5	0.0
*P. aeruginosa*	14.0 ± 0.1	14.0 ± 0.2	0.0
*V. fluvialis*	16.0 ± 0.4	22.0 ± 0.6	0.0
*V. damsela*	20.0 ± 0.2	24.0 ± 0.2	0.0
*B. cereus*	0.0	10.0 ± 0.3	0.0
*S. typhimurium*	16.0 ± 0.2	20.0 ± 0.3	0.0

The data are represented as mean ± SD in mm of inhibition zone demonstrated, contrasted utilizing ANOVA, with a significance level (*p*-value) ≤ 0.05.

**Table 7:** MIC values of the ERF against selected pathogens.

Pathogens	MIC (mg/mL)
*S. aureus*	10.0 ± 0.06
*E. faecalis*	12.5 ± 0.02
*P. aeruginosa*	10.0 ± 0.01
*V. fluvialis*	2.5 ± 0.05
*V. damsela*	1.5 ± 0.02
*S. typhimurium*	5.0 ± 0.01
*A. fumigatus*	1.0 ± 0.02
*A. niger*	1.75 ± 0.06
*A. flavus*	1.25 ± 0.01
*A. terreus*	0.75 ± 0.01

**Table 8:** Combined activity (Inhibition zone (mm)) of ERF with Amoxicillin/clavulanic against different *V. damsela* (10^−6^ CFU/mL).

Amoxicillin/Clavulanic & ERF	Inhibition Zone (mm) of *V*. *damsela* (10^−6^ CFU/mL)
Amoxicillin/clavulanic (20/10 mcg)	24.0 ± 0.2
ERF (250 µg/mL)	20.0 ± 0.2
ERF (500 µg/mL) + Amoxicillin/clavulanic (20/10 mcg)	26.0 ± 0.2
ERF (750 µg/mL) + Amoxicillin/clavulanic (20/10 mcg)	26.9 ± 0.2
ERF (1000 µg/mL) + Amoxicillin/clavulanic (20/10 mcg)	28.0 ± 0.1

## Data Availability

Not applicable.
